# The role of clinical and sonographic assessments in pre-operative evaluation of patients with splenic injuries in a resource-limited economy

**DOI:** 10.4314/ahs.v23i1.83

**Published:** 2023-03

**Authors:** Aloysius U Ogbuanya, Stanley Nnamdi Chinedu Anyanwu

**Affiliations:** 1 Department of Surgery, Alex Ekwueme Federal University Teaching Hospital, Abakaliki (AEFUTHA), Ebonyi State, Nigeria; 2 Department of Surgery, Nnamdi Azikiwe University Teaching Hospital, Nnewi, Anambra State, Nigeria

**Keywords:** Preoperative evaluation, laparotomy, spleen

## Abstract

**Background:**

Splenic trauma has been recognized as the most common cause of preventable deaths amongst trauma patients. Due to paucity of modern diagnostic imaging facilities in our setting, determination of the error rates and role of the simple, available diagnostic approaches are worthwhile and relevant to the practice of general surgery.

**Objectives:**

The aim was to determine the role and diagnostic accuracy of clinical and sonographic assessments of splenic injuries.

**Methods:**

This was a prospective study of the value of pre-operative clinical and sonographic assessments of patients with splenic injuries in our setting.

**Results:**

A total of 111 patients with abdominal trauma were evaluated. Of these, splenic injuries were confirmed in 75 patients intra-operatively, mainly from blunt trauma. Of the 97 cases diagnosed by clinical method, 66(68.0%) were confirmed by intraoperative findings. Similarly, of 86 sonographic diagnoses of splenic injuries, 61 (70.9%) truly had splenic trauma. Sensitivity for sonographic and clinical assessments was 84.7% and 78.9% respectively. False positive and negative rates for clinical (27.3% versus 44.1%) and ultrasonographic (29.1% versus 40.0%) assessments were high.

**Conclusions:**

Majority of splenic injuries were due to blunt abdominal trauma. The two diagnostic methods showed high sensitivity, but performed poorly for other validity tests.

## Introduction

Globally, trauma has emerged a leading cause of morbidity and mortality in the young population^1^,^2^. Recently, published data showed that trauma-related disability adjusted life years (DALYS) has declined in high human development index (HDI) countries, but in most low and middle-income countries (LMICs), rates have continued to increase^1^,^2^,^3^. Available clinical data showed that LMICs often do not have comprehensive urban speed limit laws, seat-belt laws, motorcycle helmet and /or drink-drive laws and where the laws exist, there is poor enforcement^2^. The effects of the inefficiency of these laws have significantly affected the rates and severity of splenic injuries in LMICs because majority of the injuries arise from road traffic injuries^2^,^4^,^5^. It has been reported that spleen is the most frequently injured organ in blunt abdominal trauma and that splenic injuries account for 25% of all solid abdominal organ injuries^4^,^5^,^6^. In patients with splenic trauma, mortality rates ranging between 7-18% have been quoted^4^. The spleen is critical in regulating immune homeostasis and hemopoiesis through its ability to link innate and adaptive immunity, and in protecting against infections^5^,^7^–^9^.

Against this backdrop, emphasis has therefore, shifted towards non-operative management (NOM) of splenic injuries and splenic salvage operations since the recognition of OPSI several decades ago^5^–^8^,^10^–^12^. Importantly, the change in the surgical principle that favors splenic salvage was enabled due to the emergence of modern imaging diagnostic facilities like computerized tomography (CT) scan as investigative tool for serial evaluation of solid organ injuries^6^,^13^. In the developed economy, the success of NOM of splenic injuries has been widely reported, though patients' selection and active, serial clinical evaluation of patients receiving NOM must be emphasized^5^,^6^,^12^. Implementation of NOM in developing nations faces several challenges and this has limited the clinical experience, practice and volume of available published data on this subject in our environment^4^,^5^,^6^. Late presentation, paucity of diagnostic imaging facilities, unaffordability of the modern diagnostic tests (CT scan or magnetic resonance imaging-MRI) when present and overall deplorable heath-seeking behaviors of patients in developing nations have hampered the growth of NOM in our environment^5^,^6^,^12^,^13^.

In the classic form, massive haemorrhage from traumatized spleen can be life-threatening and urgent splenectomy may be expedient to save life^4^,^5^,^6^,^12^. In our environment with dearth of modern diagnostic and therapeutic facilities like CT, MRI and laparo-endoscopic services, coupled with limited workforce, combined sonographic assessment and timely, detailed/span> clinical assessment of patients with suspected splenic injuries by a dedicated surgical team must be emphasized to recognize dangerous injuries that will benefit from early operative treatment. Facilitated consultation and accelerated clinical decision-making in the setting of splenic trauma is especially gainful for a surgeon practicing in rural or semi-urban settlement where advanced imaging facilities like CT scan and MRI are scarcely available.

From the foregoing, there is need to determine the error rates of clinical and ultrasound assessments of patients with suspected splenic injuries in our locality. It is hoped that knowledge of the diagnostic accuracy of these diagnostic tools may perhaps, form a template for local surgeons to develop management guidelines specific to developing economy. If properly harnessed, follow-up studies can be undertaken to design local strategic guidelines in a manner that patients can be assigned into groups based on the clinical grades or degree of splenic injuries with the primary purpose to select patients for either early operative treatment or NOM followed by active serial evaluation. The aim of this study was to determine the diagnostic value of pre-operative clinical and sonographic evaluation in the diagnosis of splenic injuries, using intra-operative findings as the reference standard. The study also documented the incidence, etiological spectrum and early treatment outcomes of splenic injuries at district hospitals in our setting.

## Patients and Methods

### Design and setting

This was a prospective study of patients with splenic injuries managed at three selected district hospitals in southeast Nigeria from January 2016 to December 2020.

### Subjects

Initially, all patients with clinical diagnosis of abdominal injuries were seen and examined. However, only patients who had radiological assessment and operative treatment in addition to detailed pre-operative clinical evaluation were included in this study. Those who had clinical and radiological diagnosis of abdominal injuries, but were managed by non-operative management (NOM) were excluded. Those who refused to give consent, were too ill to give consent (or next of kin not available to give a consent) or those who died before operative treatment were excluded.

### Procedure

Each patient was evaluated clinically at the accident and emergency department (A/E) of the hospitals. The socio-demographic and detailed clinical data were extracted from each patient and recorded in a proforma. At A/E, initial care followed the advanced trauma life support (ATLS) principles including blood, fluid and electrolyte replacement. Detailed clinical evaluation concerning the mechanism of injury, site of injury, abdominal findings, hemodynamic state, blood transfusion requirement and suspected multiple intra-abdominal injuries were sought and recorded. Additionally, pulse rates at presentation and postoperatively, co-existing medical conditions, presence of delay before presentation, pre-hospital care and hourly urine output were recorded.

Pre-operatively, clinical assessment and ultrasound evaluation were performed for each patient. In those with sonographic features of splenic trauma, injuries were graded I to V using the organ injury scaling of American Association for surgery of Trauma (AAST)^3^,^4^,^12^. The ultrasound examinations were performed by two specialist radiologists and three experienced radiographers. All preoperative diagnoses were later correlated with intraoperative assessment grading (performed according to American Injury Severity-AIS^12^) system which formed the reference standard for both the clinical and ultrasound evaluation. Those with stable haemodynamic parameters or delayed presentation were sent for abdominal CT scan.

At time of laparotomy, associated injuries and type of operative treatment (splenic salvage or total splenectomy) were noted and recorded. Early postoperative complications and number of deaths were recorded. In the early perioperative period (< 24 hours), oxygen saturation, systolic blood pressure and respiratory rates were recorded. Furthermore, length of ICU admission and presence of sepsis or intra-abdominal collections were recorded.

### Data analysis

Data analysis was done using Statistical Package for Social Sciences (SPSS) software version 22.0 (IBM, Chicago, IL, USA, 2015). Data were presented as mean, standard deviation, percentages and tables. Confidence interval was calculated at 95% level and significance at 5% probability level (p<0.05).

### Ethical Approval

The protocol for this study was approved by the research and ethics committee of the three hospitals before commencement of the study.

## Results

### Patients' characteristics

During the study period, combined pre-operative clinical and ultrasonographic assessments revealed that 132(68.0%) out of 194 patients with abdominal trauma required urgent operative management. However, only 111(84.1%) of the 132 patients met the inclusion criteria and were further evaluated. There were 89(80.2%) males and 22(19.8%) females with a male to female ratio of 4:1. Their ages ranged from 10 to 74 years with a mean of 34.6+SD17.32. Approximately four-fifth (89, 80.2%) were 50 years and below. The vast majority (86,77.5%) reside in rural and semi-urban areas; the remaining 25(22.5%) were urban dwellers. Majority (52,46.9%) were traders followed by farmers (30,27.0%), artisans (11,9.9%), civil servants (10,9.0%) and others (8,7.2%).

### Mechanism of injuries and clinical presentation

Out of the 111 patients with clinical and sonographic evidence of abdominal trauma who required emergency laparotomy, intra-operative findings showed that only 75(67.6%) truly sustained splenic injuries while the remaining 36(32.4%) had injuries involving other abdominal viscera. The relative frequencies of the various causes of injuries are shown below (Table1).

**Table 1:** Mechanism of injury

Mechanism of injury	Splenic injury	No splenic injury	Total (%)
Road traffic accident	44	16	60(54.1)
Fall from Height	16	4	20(18.0)
Gunshot	10	14	24(21.6)
Domestic violence	1	1	2(1.8)
Assault and Battery	3	1	4(3.6)
Industrial injury	1	0	1(0.9)
Total	75	36	111(100.0)

Of the 75 patients with confirmed splenic trauma, 54(72.0%) had blunt abdominal trauma (BAT) and the remaining 21(28.0%) sustained penetrating abdominal trauma (PAT). Majority (37,68.5%) of BAT cases were due to RTA while nearly half (10,47.6%) of the PAT cases were due to gunshot injuries. Forty-eight of the 111 cases presented in shock. Of these, 44(91.7%) had splenic injuries while the remaining 4(8.3%) had other injuries. Put differently, shock was present in 44(58.7%) of the 75 patients with splenic injuries and in 4 (11.1%) of the 36 patients with other non-splenic abdominal injuries.

Out of the 55(49.5%) cases that needed urgent blood transfusion at presentation, 49 had splenic injuries and 6 had other injuries giving rise to pre-operative transfusion requirements of 65.3% for splenic injuries and 16.7% for other abdominal visceral injuries. There was associated haemothorax, lower rib fractures and gastric laceration in 5(6.7%), 12(16.0%) and 2(2.6%) of the 75 patients with splenic trauma.

### Diagnostic accuracy of pre-operative clinical and sonographic assessments

Pre-operatively, clinical assessment showed that, of the 111 cases studied, 77 had diagnosis of unequivocal splenic injuries, 20 patients received equivocal diagnosis (suspicious of splenic injuries) while the remaining 14 patients were diagnosed with ‘no splenic trauma’. However, intraoperative evaluation confirmed that only 56(72.7%) of 77 clinically diagnosed splenic injuries were truly so. The diagnostic role of pre-operative clinical and ultrasonographic assessment using intra-operative findings as reference standard is shown below (Table 2).

**Table 2:** Intraoperative assessment as reference for clinical and sonographic tests

Assessment Tool	Frequency	Intraoperative assessment (Standard Test)	
		True splenic injury	No splenic injury
**Clinical Assessment:**			
Splenic injury	77	56	21
Suspicious of splenic trauma	20	10	10
No splenic injury	14	9	5
Total	111	75	36
**Sonographic Assessment:**			
Splenic injury	86	61	25
No splenic injury	25	14	11
Total	111	75	36

Utilizing the intraoperative findings (using American Injury Severity-AIS grading system) as reference standard, the diagnostic concordance with ultrasonographic grading was determined for each injury grade (Table 3).

The true grades of the 70 wrong ultrasonographic gradings were determined by intraoperative assessment (Table 3b).

**Table 3b:** Intraoperative grades of the missed/ wrong sonographic grading

Wrong/Missed grading		Reference standard (intraoperative grading)					
by Ultrasonography (n=70)		Intraoperative Grades/ No injury					
	Frequency(n=70)	No Injury	I	II	III	IV	V
Grade I	2	1	-	1	-	-	-
Grade II	6	5	1	-	-	-	-
Grade III	23	9	-	4	-	8	2
Grade IV	25	5	-	1	4	-	15
Grade V	4	1	-	-	-	3	-
No splenic injury	10	-	6	2	1	1	-
Total	70	21	7	8	5	12	17

When tables 3a and 3b are combined, intraoperative assessment showed that there were 8, 10, 11, 23 and 23 grade I, grade II, grade III, grade IV and grade V splenic injuries respectively. Of these, ultrasound correctly determined injury grades in 26 cases. Of the 36 cases of “no splenic injury”, ultrasound correctly identified 15. Overall, only 41 of the 111 abdominal trauma cases were correctly typed by the ultrasound method, giving an overall concordance of 36.9%.

**Table 3a:** Correlation of sonographic and intra-operative grading

Sonographic grading	Frequency	Intra-operative grading		concordance (%)
		Correct grade	wrong grade	
Grade I	3	1	2	33.3
Grade II	8	2	6	25.0
Grade III	29	6	23	20.7
Grade IV	36	11	25	30.6
Grade V	10	6	4	60.0
No splenic injury	25	15	10	60.0
Total	111	41	70	36.9

Furthermore, the validity test results for both pre-operative clinical and ultrasonographic assessment were computed as shown below (Table 4).

**Table 4:** Validity test results

Validity test (%)	Assessment Tool	
	Preoperative clinical test	Preoperative sonographic test
Sensitivity	78.9	84.7
Specificity	47.5	35.9
False Positive Rate	27.3	29.1
False Negative Rate	44.1	44.0
Positive Predictive Value	72.7	70.9
Negative Predictive Value	55.9	56.0
Overall Diagnostic Accuracy	58.3	67.

### Surgical treatment and anaesthetic assessment

There were 29(26.1%), 60(54.1%) and 22(19.8%) patients with ASA III, ASA 1V and ASA V classes. However, 48(64.0%) of 75 patients with splenic trauma had ASA IV to V scores while only a third (12, 33.3%) of the 36 patients with non-splenic injuries had high ASA scores (ASA IV-V). Of the 75 confirmed splenic trauma cases, 62 (82.7%) had splenectomy while the remaining 13(17.3%) received splenic salvage procedure(splenorrhaphy).

### Outcomes of surgical treatment

Wound infection was the most common complication, occurring in 14(18.7%) and 9(25.0%) of those with splenic and other visceral trauma cases respectively. Six deaths (8.0%) occurred in the splenic injury patients, but in the 36 non-splenic trauma cases one death (2.8%) occurred. The pre-, intra- and post-operative characteristics of mortality cases in the splenic trauma patients are shown below (Table 5).

**Table 5:** Clinical parameters in the six death cases

Clinical parameters	Mortality cases (n=6)					
	1	2	3	4	5	6
**Pre-operative parameters:**						
Systolic BP (mmHg)	110	120	108	110	80	60
Pulse Rate (b/min)	102	96	112	99	124	154
Delayed presentation	+	+	+	+	+	+
Age (years)	62	61	41	24	26	32
Units of blood transfused	6	4	7	4	6	7
One or more comorbidities	+	+	-	+	-	+
Multiple associated injuries	+	+	+	+	-	-
Hourly urine output (ml)	15	20	15	10	15	25
**Intra-operative parameters:**						
Intra-peritoneal fecal soilage	+	+	-	+	-	+
Operative procedure	TS	TS	TS	TS	PS	TS
**Post-operative parameters:**						
Systolic BP (mmHg)	110	106	114	80	76	100
Pulse Rate	96	110	92	121	128	116
Sepsis	+	+	+	-	+	+
Oxygen saturation (%)	68	83	97	96	84	92
Intra-abdominal collection	+	+	+	-	-	+
Respiratory Rate	26	34	30	28	22	40
ICU admission > 5 days	+	-	+	-	-	-

ICU= intensive care unit; BP= Blood pressure; ml=millimeter; TS= total splenectomy; PS= partial splenectomy (splenic salvage)

## Discussion

Published clinical studies indicate that abdomen is the third most injured region in trauma patients and splenic rupture is the most frequent cause of preventable deaths in patients with injuries^4^,^5^,^6^. Therefore, pre-operative assessment of trauma patients using clinical and sonographic methods to achieve accurate diagnosis of splenic involvement remains an indispensable tool in the overall work up of spleen-injured patients especially in poor resource setting like ours. Indeed, the evaluation of the quality of diagnostic services for splenic injuries in our environment cannot be over-emphasized. The high rates of late presentation, limited workforce and therapeutic facilities, ignorance and poverty with consequent high morbidity and mortality rates further emphasize the imperativeness of a system-wide approach to diagnosis and treatment of patients with suspected splenic trauma in emerging economy like Nigeria.

In this study, the patients' population comprised predominantly young and middle-aged persons who were mostly rural/semi-urban residents. There was also male preponderance with majority being traders and farmers. Findings from previous studies done elsewhere conform with the above report^4^–^6^,^12^,^14^. It has been reported that trauma rates are generally more in males and younger individuals due the fact that these groups are generally more exposed to trauma and violence from increased human movements, possession of firearms and higher involvement in illicit acts like drug abuse, alcoholism, kidnapping, cultism, insurgency and wars^24^,^25^,^27^–^30^.

Not withstanding the fact that this study was carried out in district hospitals with sub-optimal facilities for managing trauma patients, we determined that 67.7% of patients undergoing emergency laparotomy for trauma sustained splenic injuries during the period of study. These findings conformed with report from Nigeria^14^–^18^, central Africa1, India^19^, South Africa^20^, Ethiopia^21^ and Poland^22^. It has been established that despite being protected under the bony ribcage, the spleen is vulnerable to abdominal trauma and remains the most frequently injured solid abdominal viscera in blunt abdominal trauma^4^–^6^,^13^,^18^. In summary, spleen is the most frequently injured intra-abdominal solid organ in most series especially in Africa where some patients have splenomegaly due to haemoglobinopathies and chronic parasitic infestations from malaria, schistosomiasis and visceral Leishmaniasis^12^,^13^,^23^.

In northern Tanzania, Ntundu and colleagues reported on a large series of 136 patients with abdominal trauma and found that splenic trauma accounted for 91.7% of patients who sustained BAT, despite exclusion of those managed conservatively1. The reason adduced included greater proportion of patients with BAT compared to PAT1. In this series, however 72.0% of patients with splenic trauma had BAT. The explanation for higher incidence of PAT (28.0%) among the spleen-traumatized patients in our study compared to values quoted in Tanzania1 may be partly explained by the findings of previous investigators that rising rates of famers-herders clashes, armed robbery attacks, political thuggery, kidnapping and cultism activities are on the increase in southeast Nigeria in recent time^24^,^25^.

The validity test results observed in this study showed mixed pattern with pre-operative clinical assessment performing better than sonographic results in some areas and vice versa for other validity tests (Table 4). Overall, clinical assessment was more specific than sonographic test, but less sensitive for splenic trauma. This means that when splenic injury is present, ultrasound test is more reliable, but in the absence of splenic trauma, clinical assessment has better ability to unequivocally exclude the diagnosis. Generally, the hallmark of splenic trauma is haemorrhage that leads to progressive hemoperitoneum which is easily detected by ultrasound as echo-rich fluid in the pelvic, subphrenic and subhepatic spaces^3^,^5^,^12^,^26^. This partly explains the higher sensitivity of sonographic assessment compared to clinical method. However, higher volume of hemoperitoneum is required before it can be detected by clinical method thereby reducing its clinical reliance. It has been shown that intraperitoneal fluid as low as 300ml can be detected by ultrasound, but higher volumes are required before free intraperitoneal fluid can be elicited clinically^27^,^28^.

Importantly, hemoperitoneum is not specific to splenic injuries and substantial, life- threatening intra-peritoneal haemorrhage has been reported in the setting of liver, mesenteric, renal and vascular (inferior vena cava, aorta, portal vein, iliac) injuries of the abdomen^12^,^13^,^26^. In practice, however, clinical parameters like positive peritoneal tap at the left hypochondrium, local bruising, left hypochondriac tenderness or guarding and left lower rib fractures are associated with splenic trauma; when present, these sharpen the clinical picture and may contribute to the higher specificity of clinical assessment compared to sonographic test. Classic signs such as Kehr's sign (left shoulder tip pain) and Balance's sign (non-shifting left flank dullness indicating a peri-splenic haematoma) though often not present, but when elicited clinically, are specific for splenic trauma^27^,^28^.

The high false positive rate (FPR) and false negative rate (FNR) for both diagnostic methods are worrisome and major clinical decisions must be made in the context of combined clinical and radiological assessments. Sources of false positive diagnosis for sonographic test were haemorrhage from other solid organs or mesenteric /vascular bleeding and trauma of contiguous structures (pancreas, stomach, left colon) with resultant para-splenic haematoma. Similarly, false positive diagnosis of splenic injury from clinical standpoint may be due to left hypochondriac mass and tenderness from other abdominal visceral injuries and isolated left lower rib fractures without accompanying splenic trauma. In summary, FPR is higher with sonographic test than clinical assessment due to more differential diagnoses of hemoperitoneum and left upper abdominal mass shadows. The danger with high FPR lies with over-treatment of patients who otherwise do not have indication for operative management or application of invasive diagnostic or therapeutic procedures like angio-embolization or diagnostic peritoneal lavage (DPL).

The FNR of 44% recorded for both diagnostic methods were contributed by the presence of extra-abdominal injuries like head injury that overshadowed the abdominal signs, slowly evolving hemoperitoneum from low grade splenic injuries and missed diagnosis due to lack of classic history and physical findings. Unfortunately, false negative diagnosis for splenic trauma especially in hemodynamically unstable patients may lead to delay in operative treatments and increase in the percentage of preventable deaths. Indeed, the danger lies with the delay rather than in the operation in patients with splenic injuries especially those with significant ongoing haemorrhage.

From the point of view of global best practices, FPR and FNR in patients with splenic trauma should be reduced to the barest minimum to avoid both overtreatment and neglected trauma cases. In the resource-poor economy like ours, avoidable deaths from delayed treatment and negative laparotomy from unmerited OM have continued to be high due to lack of modern diagnostic imaging studies like CT scan and MRI in many communities in LMICs^3^,^4^,^5^,^12^. Though the sensitivities and positive predictive values for both pre-operative tests were above average in this study, the two diagnostic approaches performed poorly in the other validity tests reported (Table 4). These observations therefore, render both diagnostic tests less reliable when considered alone and as a matter of necessity, demands a mandatory combined diagnostic approach in all cases of suspected splenic trauma. These results overlapped with reports from referral centres in Ibadan and Gombe, both in Nigeria^3^,^12^.

In the current era of shift towards NOM for splenic injuries, accurate pre-operative diagnosis, grading and selection of patients have become increasingly recognized as the anchor sheath in planning management strategy^5^,^6^,^12^. False positive diagnosis with overzealous OM exposes the patients to the adverse aftermaths of laparotomy like surgical site infection, iatrogenic injuries, sepsis, incisional hernia, atelectasis or even death^5^,^6^,^13^,^29^. Aside avoiding unnecessary OM, accurate pre-operative diagnosis and classification of splenic injury enables splenic preservation in properly selected patients^3^,^12^,^26^,^30^. The more recent understanding that splenectomy predisposes patients to immunological alterations that can lead to infections by encapsulated organisms and in extreme cases, overwhelming post-splenectomy infection (OPSI) has rekindled interest in NOM and splenic salvage procedures (when operative management becomes inevitable)^5^,^6^,^8^,^13^,^30^.

Currently, contrast-enhanced CT scan of the abdomen is the modality of choice for diagnosis and evaluation of splenic injuries in patients that are hemodynamically stable^26^,^31^,^32^. Published data from many series have reported superior validity test results for CT compared to ultrasound in preoperative assessment of patients with splenic injuries or blunt abdominal trauma^26^,^31^,^32^. In Iran, a multicenter study involving 68 patients with splenic trauma revealed variable CT performance for various grades^31^ . Specificity was 90.3%, 38.7% and 22.6% for grade II, grade III and grade IV respectively while sensitivity was 51.4%, 94.5% and 100.0% for grades II, III and IV respectively^31^. More impressive findings were reported in Pakistan by Maria and colleagues following CT assessment of 125 patients with blunt abdominal trauma^32^. The authors quoted sensitivity, specificity, negative predictive value and positive predictive value of 100.0%, 91.7%, 100.0% and 85.4% respectively^32^. The apparent better performance of CT in Pakistan compared to Iranian series may be explained by the fact that combined validity results were computed in the Pakistani study where injuries to the retroperitoneum, bowel, mesentery, liver and kidney were considered^32^. It has been shown that overall, CT has sensitivity of 100.0% for liver injuries and 86.6% for splenic trauma and for specificity, retro-peritoneum (100.0%) and kidney (93.5%) have the highest CT performance in patients with blunt abdominal trauma^32^. Despite the higher diagnostic accuracy of CT compared to ultrasound, the benefits of ultrasound in preoperative evaluation of patients with splenic trauma cannot be overemphasized especially in rural communities considering the high cost, often unavailability and higher radiation dose of CT scan^31^,^32^. Moreover, ultrasound is an important investigative tool in early assessment of both haemodynamically stable and unstable patients due to its portability, ease of execution and good diagnostic yield^26^,^31^,^32^.

In our setting with poor health infrastructure and low human development index (HDI), there is need to improve clinical expertise of surgeons and other clinicians involved in trauma management especially for the health personnel practicing in district/rural areas. The pre-operative clinical parameters of the six patients that died after operative management showed striking similarities in many clinic-pathologic parameters (Table 5). For the six mortality cases, overlapping results were obtained for injury-arrival interval, multiple transfusion requirement, presence of comorbid illnesses, multiple associated injuries, hourly urine output and oxygen saturation.

## Limitation

This study involved only patients managed operatively and therefore the role of clinical and sonographic assessments for other grades of splenic injuries managed by NOM was not ascertained. Moreover, the follow up period was very short. The value of CT scan for splenic injuries was not evaluated due to non-availability and paucity of expertise to interpret its findings in our setting.

## Conclusion

Majority of splenic injuries in our environment are due to BAT with RTA being the commonest cause of BAT. The sensitivity of sonographic and clinical assessment for splenic injuries are above average, but overall, both diagnostic methods proved themselves less reliable diagnostic tools in our environment. The predictors of mortality included multiple transfusion requirement, multiple associated injuries, delayed presentation, comorbidities, inadequate urine output and poor oxygen saturation postoperatively.

Furthermore, with more robust analytical cross-sectional studies and long patients follow up, a local protocol can be developed to risk-stratify patients pre-operatively using clinical and radiological parameters. The high-risk patients can be selected early enough using clinical assessment and more vigorous treatment and monitoring performed to reduce mortalities.
